# Radiostereometric Analysis Allows Assessment of the Stability and Inducible Displacement of Pelvic Ring Disruptions during Healing: A Case Series

**DOI:** 10.3390/jcm9113411

**Published:** 2020-10-24

**Authors:** Andreas Ladurner, Stuart A. Callary, Aniruddha Mitra, Mark Rickman, Dominic Thewlis, Lucian B. Solomon

**Affiliations:** 1 Department of Orthopaedics and Trauma, Royal Adelaide Hospital, Adelaide, SA 5000, Australia; andreas.ladurner@kssg.ch (A.L.); stuart.callary@sa.gov.au (S.A.C.); mark.rickman@sa.gov.au (M.R.); 2 Centre for Orthopaedic and Trauma Research, The University of Adelaide, Adelaide, SA 5000, Australia; dominic.thewlis@adelaide.edu.au; 3 Department of Orthopaedics and Trauma, Warrnambool Hospital, Warrnambool, VIC 3280, Australia; mitraani@gmail.com

**Keywords:** pelvic ring injury, radiostereometric analysis (RSA), fracture

## Abstract

There is currently no accurate data on fracture displacement during the rehabilitation of pelvic ring injuries. This study investigated the use of radiostereometric analysis (RSA) in assessing the stability of C1 pelvic ring injuries stabilised with a posterior plate and an anterior external fixator. Six patients, instructed to weight-bear as tolerated after surgery, were reviewed at 2, 4, 6, 12, 26, 52 and 104 weeks. The external fixators were removed at 6 weeks. Outcomes, including the Iowa Pelvic Score (IPS), and complications were recorded. Fracture stability was assessed using measurements on plain radiographs and RSA. All patients progressed to full weight-bearing without support within 6 weeks. At 104 weeks, the IPS was excellent in four patients, good in one patient and fair in one patient. Plain radiographs showed that all fractures were well reduced, and no loss of reduction occurred over time. By contrast, RSA measurements identified displacement in all cases. The maximum three-dimensional (3D) displacement at any time point in each patient ranged from 2 to 10 mm. Two patients with the largest displacement over time had the lowest IPS. RSA also demonstrated displacements above the currently defined normal threshold through the ‘un-injured’ sacroiliac joint in the same two patients, suggesting a subtle C2 injury, missed at initial assessment. This study demonstrates the limitations of plain radiographs in assessing pelvic fracture stability and displacement during healing, and the potential of RSA to monitor more accurately the effects of stabilisation and weight-bearing on fracture stability.

## 1. Introduction

**Figure 1:**
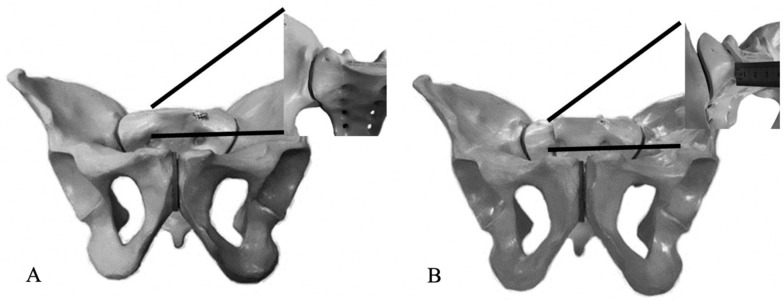
The projection of the pelvic ring on an outlet view can fail to identify a 1 cm vertical displacement as proven by the inset images. (**A**) Anatomically reduced injury, and (**B**) injury mal-reduced by 1 cm.

Type C pelvic ring disruptions are rare but severe injuries [1,2], rotationally and vertically unstable [3], that commonly lead to significant long-term functional limitations for the patient [4,5,6]. Current literature on the early rehabilitation of severe pelvic ring injuries is limited and mostly recommends non weight-bearing or touch weight-bearing for up to three months [7,8,9]. Immediate postoperative weight-bearing following surgical stabilisation of these injuries is believed by many to risk loss of reduction and subsequent malunion or non-union. However, prolonged periods of immobilisation are also associated with significant morbidity [10]. To date, there is no data on the magnitude of fracture displacement caused by weight-bearing after stabilisation of these injuries. Loss of reduction and fixation failure in type C pelvic ring injuries has so far only been assessed on plain radiographs and defined as > 1 cm of displacement [11,12,13]. Due to the complex three-dimensional (3D) shape of the pelvic ring, fracture displacement can be very difficult to assess on plain radiographs, reflected by this large threshold [11,14,15,16]. Additionally, the pelvis will likely have a slightly different position on the X-ray at each examination and assessing fracture displacements on radiographs does not take into account translations outside the plane of the X-rays. This may explain in part why some pelvic ring disruptions, thought to have been well reduced and healed without loss of reduction (Figure 1), have less than satisfactory patient-reported outcomes. 

The ability to provide accurate objective data to investigate the effect of weight-bearing on the stability of surgically stabilised pelvic ring disruptions has the potential to optimise operative stabilisation techniques as well as post-operative mobilisation guidelines. Guidelines based on such data could minimise the risk of complications related to either unnecessary immobilisation or excessively aggressive mobilisation following surgery. Effective post-operative management guidelines could lead to improved clinical outcomes and fewer complications.

The biggest limitation on the development of evidenced-based fixation techniques and rehabilitation protocols for severe pelvic ring disruptions is the inability to measure stability accurately on plain radiographs in the early postoperative period [11,14,15,16]. Although computed tomography (CT) scans are capable of detecting small fracture migrations, CT’s cannot be used repeatedly due to radiation exposure and cannot typically be used under weight-bearing load conditions. By contrast, radiostereometric analysis (RSA) can accurately measure fracture migration [17,18,19], can be performed at regular intervals without exposing patients to higher radiation doses than regular radiographs [20], and differentially loaded RSA (DL-RSA) can be performed under weight-bearing loads [19,20]. In a subgroup of tibial plateau fractures, DL-RSA measurements have shown that instruction to ‘weight-bear as tolerated’ resulted in less than 1 mm fracture migrations that were elastic in nature throughout fracture healing [19]. 

To date, there is no published data on the use of RSA to investigate pelvic ring injuries. Therefore, the aim of this study was to investigate the feasibility of RSA and DL-RSA to investigate the stability of type C1 pelvic ring injuries in a case series of patients that had surgical stabilisation with a posterior plate and an anterior external fixator and were allowed immediate unrestricted postoperative weight-bearing. 

## 2. Experimental Section

A prospective case series of patients were investigated at our institution, a public level I trauma centre. Our institution’s Ethics Committee approved the study and each patient provided written informed consent to participate (#060621, 31 July 2006).

All patients treated for pelvic ring injuries over a period of 18 months were considered for this study. Inclusion criteria included any patient willing to participate with a Tile C1 pelvic ring injury [21,22] where the posterior pelvic complex injury was amenable for plating and the anterior pelvic complex injury was amenable for definitive treatment with a pelvic external fixator. The indication for posterior pelvic ring plating was an unstable posterior ring injury with displacement, as un-displaced posterior ring fractures were commonly considered for sacroiliacal screw fixation. A pelvic external fixator was applied with comminuted fractures of the pubic rami, where open stabilisation was not necessary, while simple fractures were more commonly stabilised using percutaneous screw fixation. Exclusion criteria were sacral fractures medial to the sacral foramina, injuries to the anterior complex involving symphyseal diastasis (requiring increased anterior stabilisation time) and associated injuries that could have precluded patient mobilisation after pelvic stabilisation. 

**Figure 2:**
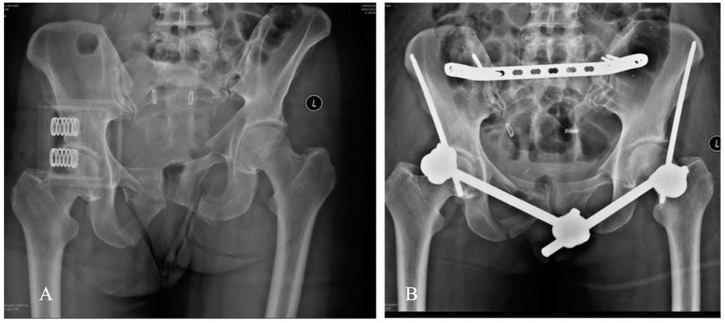
(**A**) AP pelvis X-ray of Patient 1 pelvic ring injury. (**B**) AP pelvic X-ray of the same pelvis after reduction and stabilised with a posterior plate and an anterior subcristal external fixator.

Diagnoses were made on plain radiographs (antero-posterior (AP) pelvis, inlet and outlet views) and CT scans in all cases. All cases were temporarily stabilised in the Emergency Department with a pelvic binder and trans-femoral skeletal traction. Definitive fixation was performed by a single surgeon (L.B.S.) at a mean of 7 days (range 5 to 14 days) after injury. All patients underwent reduction and internal fixation of the posterior pelvic complex, with a pre-contoured 4.5 mm narrow Locking compression plate (LCP, Synthes, Paoli, PA, USA) inserted through 2 vertical posterior incisions. After internal fixation, a minimum of 6 tantalum markers (1 mm diameter, RSA Biomedical, Umeä, Sweden) were inserted into the sacrum and each iliac wing using a spring-loaded piston, matching previous recommendations [23]. The anterior pelvic complex was stabilised with a subcristal external fixator [24] (Figure 2). Postoperatively, all patients were encouraged to weight-bear as tolerated as soon as they regained leg control and were advised to use crutches for 6 weeks. 

All patients underwent plain radiographs and RSA imaging on day 2 postoperatively for baseline assessment. After discharge, patients were followed-up at 2, 4, 6, 12, 26, 52 and 104 weeks postoperatively, including conventional AP, inlet and outlet radiographs and RSA imaging. Radiostereometric analysis examinations consisted of three sets of radiographs: supine (pre-load), weight-bearing as tolerated while standing, and again, supine (post-load). At the time of removal of the external fixator at 6 weeks, RSA imaging was performed pre- and post-fixator removal. A uniplanar-type RSA setup with two radiographic tubes, one ceiling-mounted (Philips Bucky Diagnost, Philips Healthcare, Andover, MA, USA) and another mobile X-ray tube (Philips Practix 8000, Philips Healthcare, Adover MA, USA), was used. The calibration cage (Cage43, RSA Biomedical, Umeä, Sweden) containing two high-resolution digital radiographic cassettes (35 × 43 cm each) was placed at a focal length of 1.6 m. All radiographs were exposed at 110 kV and 16 mAs. The image plates were digitised with an AGFA Centricity CR SP1001 processor. The DICOM images were downloaded as Tagged Image Format (TIF) images at 300 DPI resolution. Each radiographic examination was analysed using the UmRSA software (v6.0, RSA Biomedical, Umeä, Sweden). Two standard quality control parameters of RSA were applied. An upper limit of 0.35 mm for the mean error (ME) of rigid body fitting as an indicator of marker stability was used for all RSA measurements. Each rigid body had a condition number (CN) below 150, which is an indication of adequate marker spread [20].

Fracture displacement was defined as any change in translation or rotation between the ilium on the injured and uninjured side relative to the sacrum. Translations and rotations along each of the x-, y- and z-axes were measured as well as the three-dimensional (3D) displacement (i.e., the vectorial sum of x-, y- and z-axis translation). Fracture displacement over time was calculated as the change in position of the ilium relative to the sacrum at each time point in reference to the initial supine postoperative RSA examination. To measure displacement under weight-bearing load, the supine (pre-load) radiographs at each time point were used as a reference. The post-load supine examination was used to determine if displacements under load were elastic or plastic. Displacements less than 2 mm of translation and 3.6° of rotation across the un-injured sacroiliac joint (SIJ) were defined as physiological [23]. All data were expressed in a standardised right-hand coordinate system. Therefore, all left data were inverted to standardise reporting.

Reduction was assessed on the postoperative plain radiographs according to Tornetta and Matta [7,25]. We measured postoperative fracture displacement in the anteroposterior, inlet and outlet view. Reduction was graded by the maximal displacement measured on the 3 views as excellent (≤4 mm), good (4–10 mm), fair (10–20 mm) and poor (>20 mm). Loss of reduction was defined using the current criteria of fracture displacement of >10 mm on any radiograph [11,12,13]. 

All external fixators were removed 6 weeks after application. Clinical progress and complications were recorded prospectively. Clinical outcomes were assessed using the patient-reported Iowa Pelvic Score (IPS) [26,27].

Descriptive statistics included means, standard deviations, ranges and proportions. Comparative statistics included Chi-square test (where appropriate, Fisher’s exact test was alternatively applied). The confidence level for rejecting null hypotheses was set at 95% (*p*-value < 0.05).

## 3. Results

### 3.1. Demographics

Patient and injury demographics are presented in Table 1.

### 3.2. Clinical Results 

There were no intraoperative complications. Postoperatively, one patient developed a non-fatal pulmonary embolism and another patient developed a superficial pin tract infection at 4 weeks. The latter was managed with oral antibiotics until the fixator was removed 2 weeks later. 

All patients were mobilising weight-bearing as tolerated prior to discharge. Five patients were discharged directly home, whilst the sixth patient was discharged to a rehabilitation facility. All patients were full weight-bearing at 6 weeks after surgery and were only using ambulating supports when outside the house. After removal of the external fixator, two patients had ongoing pain around the posterior pelvic complex. All patients were ambulating with no detectable limp at 104 weeks follow-up.

The median IPS at 104 weeks follow-up was 84 (range 68–94). Overall, four patients had an excellent score and clinical outcome, one had good and one a fair score. Only the patients with the fair (Patient 2) and good (Patient 4) IPS score had pain after fracture healing.

### 3.3. Radiographic Results

**Figure 3:**
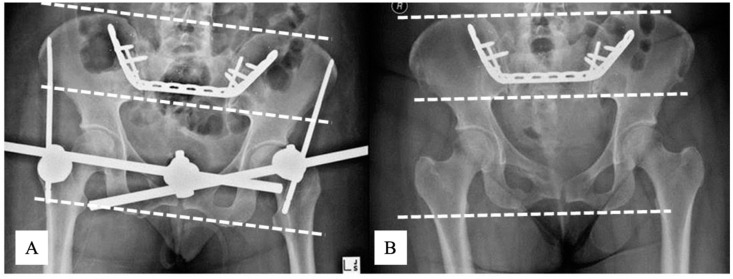
AP X-rays of Patient 2 pelvis immediately after reduction and fixation (**A**) and at 104 weeks follow-up (**B**). In this patient, RSA measured a 1 cm fracture translation during healing. Note that there are no measurable vertical translations on plain radiographs, and that the iliac bones project relatively symmetric on each image. Note the slightly different projections of the pelvis between examinations. The dotted lines correspond to a horizontal plain. AP, antero-posterior; RSA, radiostereometric analysis;

On plain radiographs, fracture reduction was assessed as “good” according to Tornetta and Matta [7,25] in all cases, as displacement measured on the AP pelvis, inlet and outlet views summarised 4–10 mm in each case. All sacral and pubic rami fractures healed with no fracture line visible on the 3-month radiographs. No loss of fracture reduction was identifiable on plain radiographs (AP pelvis, inlet and outlet views) at any time point during follow-up in any patient (Figure 3).

### 3.4. RSA

#### 3.4.1. D Fracture Fragment Displacement over Time

**Figure 4:**
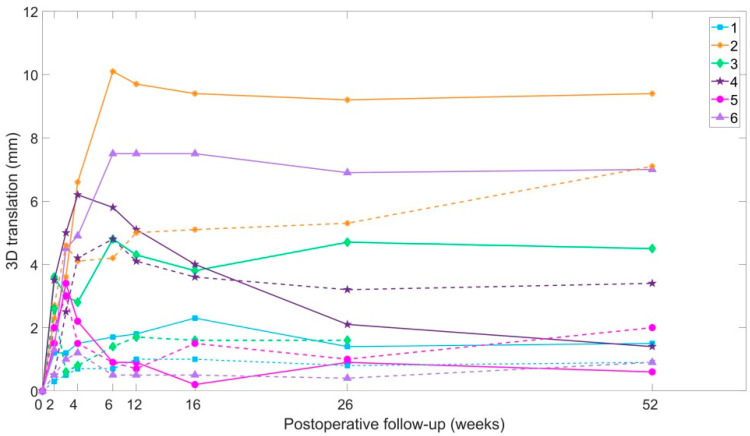
Three-dimensional (3D) displacements over time to 104 weeks. Continuous lines indicate displacements of the injured side ilium. Dotted lines indicate displacements of the non-injured side ilium.

RSA identified fracture displacement over time in all cases (Figure 4).

On the injured side, 3D displacement occurred mainly within the first 12 weeks, averaging 5.1 mm at 12 weeks. The maximum displacement at any time point in each patient ranged from 2 to 10 mm, with Patient 2 just exceeding 10 mm at 12 weeks (10.1 mm). Between 12 and 104 weeks, 3D translation of the injured side ilium relative the sacrum was <1 mm in 4 of the 6 patients, while Patients 2 and 4 showed ongoing migration.

On the uninjured side, displacement across the SIJ was less than that across the injured side, but Patients 2 and 4 exceeded the physiological threshold of 2 mm. In Patient 2, this continued after 12 weeks and resulted in a maximum displacement of 7.1 mm at 104 weeks follow-up. That displacement was measured across both posterior pelvis complexes in these patients suggests that their initial diagnosis should have been a Type C2, rather than a C1 injury. 

When the 3D displacements were broken down into each single axis, translation in the y-axis (cranio-caudal displacement) and rotation around the x-axis (flexion-extension of the ilium against sacrum) were shown to contribute the most to the overall displacement. Therefore, these two movements are also presented separately.

#### 3.4.2. Y-Axis Translation

**Figure 5:**
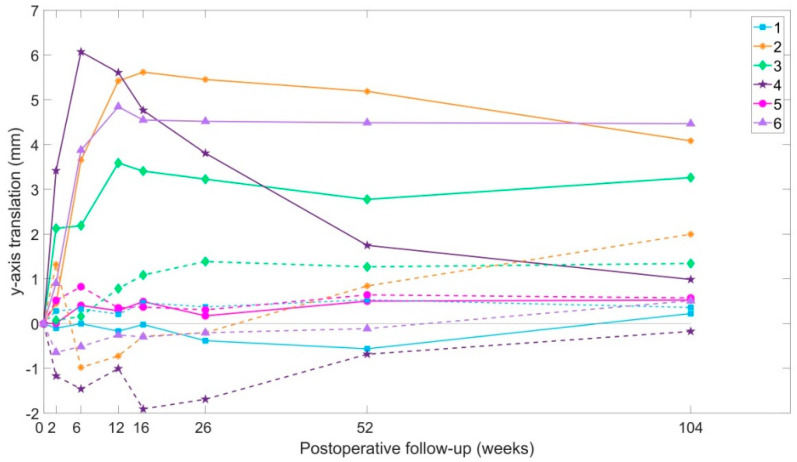
Proximal (Y-axis) translations over time. Continuous lines indicate displacements of the injured side ilium. Dotted lines indicate displacements of the non-injured side ilium.

In 4 of the 6 patients, the injured side ilium translated against the sacrum. The average cranial translation in all patients was 3.3 mm at maximum, with Patients 2 and 4 recording the highest translations. Translation of the non-injured side ilium was less than 2 mm in all cases (Figure 5). 

#### 3.4.3. X-Axis Rotation

**Figure 6:**
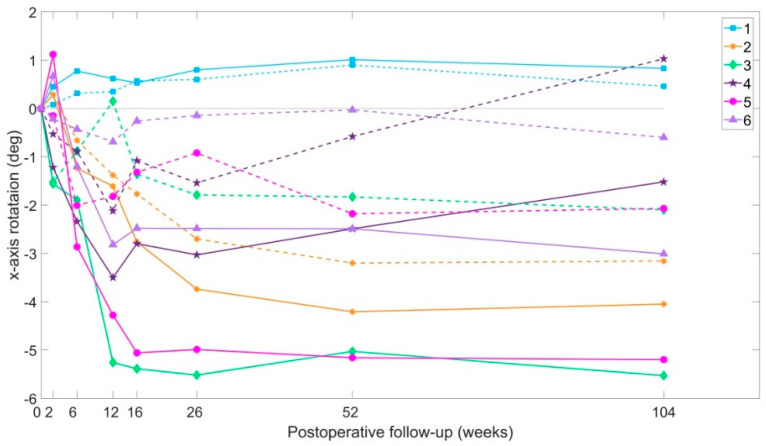
X-axis rotations over time. Positive values report anterior rotation (flexion) of the ilium against the sacrum, and negative values report posterior rotation (extension). Continuous lines indicate displacements of the injured side ilium. Dotted lines indicate displacements of the non-injured side ilium.

Mean extension of the ilium against the sacrum was 3° (range 0.8 to 5.5) on the injured side, and 1° (range 0.5 to 3.2). Patients 2 and 4 were found not to stabilise over time, as continuous rotation changes were recorded on both patients over time (Figure 6). 

#### 3.4.4. Pelvic Displacement Caused by the Removal of the External Fixator 

The RSA examination immediately after the removal of the external fixator demonstrated a 3D displacement of up to 2 mm across the injured side in Patients 2 (1.9 mm) and 5 (2.2 mm). While in Patient 2, the displacement only occurred across the injured side, Patient 5 also demonstrated a displacement of 1.9 mm on the uninjured side, as if the removal of the external fixator had released the pelvic ring into the position determined by the pre-contoured posterior plate. This finding was not correlated with any change of patient symptoms. 

### 3.5. DL-RSA Results 

**Figure 7:**
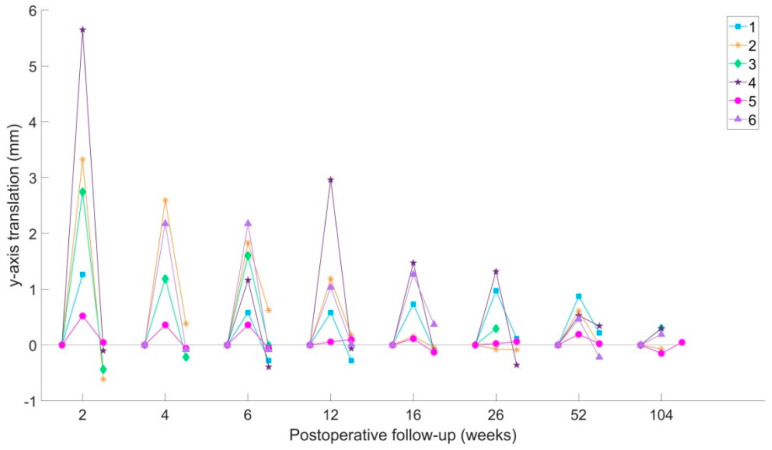
Load-dependent y-axis translations of the injured side ilium. Positive values showing cranial displacements of the ilium relative to the sacrum. Value for the supine (pre-load), standing (load) and supine after standing (post-load) position are shown for each time point.

Both y-axis translation (Figure 7) and x-axis rotation of the injured side ilium were found to be elastic in nature during the DL-RSA examinations. This finding was not affected by the presence of the external fixator. 

The amount of translation or rotation recorded between the standing and supine positions decreased over time as the injuries healed. The largest cranial translation during weight-bearing was measured in Patient 4 (5.7 mm). Mean elastic displacement of the injured side was 2.7 mm at 2 weeks after surgery, and 1.1 mm at 12 weeks. On the uninjured side, the mean cranial translation was 1.1 mm at 2 weeks. Once again, Patients 2 and 4 recorded the largest displacements under load across the injured side (3.3 mm and 5.x mm y-axis translation at 2 weeks). X-axis rotation under load across the injured side was largest at 2 weeks, mean 3° (largest in Patients 2 and 4). On the uninjured side, x-axis rotation under load was <1.8°.

## 4. Discussion

This study investigated the use of RSA to accurately assess pelvic ring displacement over time and stability under load in 6 patients with a C1 injury. Fracture displacement across the injured side was observed in all patients and decreased over time. Patients with the largest fracture displacements reported worst clinical outcomes. RSA measurements also identified subtle contralateral SIJ injuries in these two patients missed on plain radiographs and CT.

Current literature defines loss of reduction in pelvic ring injuries as a displacement >10 mm measured on plain radiographs [12,13]. No fracture displacement was measurable on plain radiographs in the six patients in this study throughout their follow-up. By using RSA, displacement across the injured side could be measured in all patients, and in one patient, the 3D displacement exceeded 10 mm at 12 weeks. 

The majority of displacement across the injured side occurred within the first 12 postoperative weeks, corresponding to fracture healing on plain radiographs. In two patients, this displacement continued between 12 and 104 weeks despite the fractures being united and appearing healed on plain radiographs. This continued displacement, that measured up to 5 mm, could have only occurred through the SIJ, demonstrating an associated SIJ injury to the sacral fracture, on the injured posterior complex side of the pelvis. Therefore, the study also suggests that the posterior fixation used in these cases might not be an efficient SIJ stabiliser and that this fixation should have been augmented with SIJ screws, or the patients mobilised less aggressively. Importantly, these two patients had the only two non-excellent IPS, and persistent posterior pain. As a result of these findings, we now supplement posterior plate fixation with SIJ screws in all C1–3 fractures at our institution. 

The timing of the removal of pelvic external fixators is controversial and likely to be multifactorial [24], and in this study, two patients experienced acute pelvic ring displacement after the removal of the external fixator. We suggest that RSA could be used as a method to optimise the timing of pelvic external fixation removal by performing RSA before and after loosening the external fixator but prior to removal of the pins. If movement is detected, then the fixator could be re-tightened, and the investigation repeated at a later stage. 

This study has several limitations. Firstly, this novel case series only included a small number of patients. Despite previous studies using small cohort numbers reporting effectiveness of RSA due to the method’s high precision [28,29,30], we acknowledge that no generalisation should be drawn from this study and that larger RSA studies are required before any evidence-based recommendations can be made. However, our results demonstrate the ability of RSA to detect translational and rotational instability of the pelvic ring during healing not visible on plain radiographs. Secondly, the study involved RSA measurements across the injured posterior pelvic complex which did not differentiate between movements that occurred through the sacral fracture and movements that occurred through the ipsilateral SIJ. It is therefore acknowledged that some of the fracture migration recorded on the injured side could represent movement that occurred through the SIJ. 

## 5. Conclusions

In conclusion, this study used RSA to measure the displacement of pelvic ring injuries throughout healing and demonstrated the limitations of plain radiographic measurements. Larger studies using RSA measurements have the potential to monitor differences between surgical treatment and rehabilitation protocols. 

## Figures and Tables

**Table 1:** Patient and injury demographics.

Number of Patients	*n*	6
**Age (years)**	Mean (Range)	34 (18–47)
**Gender**	F:M	4:2
**BMI**	Mean (Range)	23 (21–25)
**Mechanism of injury**	MVA - car vs pedestrian - car vs car - Fall from height - parachute accident - fall from horse - Work-related (hit by hay-bail)	3 2 1 2 1 1 1
**Classification of injury (Tile** [3]**)**	All patients	C1
**Associated injuries**	Chest injury Spine fractures Head injury Abdominal organ injury Superficial soft tissue injury	1 3 1 3 2
**Length of stay from initial injury (days)**	Mean (Range)	25 (16–37)

BMI, body mass index; F, female; M, male; MVA, motor vehicle accident
